# A novel immune-related long non-coding RNAs risk model for prognosis assessment of lung adenocarcinoma

**DOI:** 10.18632/aging.203772

**Published:** 2021-12-14

**Authors:** Songmei Lu, Nan Shan, Xingyue Chen, Fangliang Peng, Yiming Wang, Hao Long

**Affiliations:** 1 Department of Medical Oncology, Chongqing University Cancer Hospital, Chongqing, China; 2 Department of Gynaecology and Obstetrics, The First Affiliated Hospital of Chongqing Medical University, Chongqing, China; 3 Department of Biological Immunotherapy, Chongqing University Cancer Hospital, Chongqing, China

**Keywords:** LUAC, long non-coding RNAs, immune-related risk score model, OS

## Abstract

Background: The abundant immune-related long non-coding RNA (IRLNRs) in immune cells and immune microenvironment have the potential to forecast prognosis and evaluate the effect of immunotherapy. IRLNRs analysis will provide a new perspective for LUAC research.

Methods: We calculated the immune score of each sample according to the expression levels of immune-related genes (IRGs) and screened the survival-related IRLNRs (sIRLNRs) by Cox regression analysis. The expression levels of AC068338.3 and AL691432.2 in tissues and cell lines were confirmed by RT-qPCR.

Results: 36 IRLNRs were selected by Pearson correlation analysis. Ten sIRLNRs were significantly correlated with the clinical outcomes of LUAC patients. Five sIRLNRs were identified by multivariate COX regression analysis to establish the immune-related risk score model (IRRS). The overall survival (OS) in the high-risk group was shorter than that in the low-risk group. IRRS could be an independent prognostic factor with significant survival correlation The distributions of immune gene concentrations were different between high-risk group and low-risk group. Furthermore, we further verified that the expression levels of AC068338.3 and AL691432.2 in different LUAC cell lines and tumor tissues were lower than that in Human bronchial epithelial cell (HBE) and adjacent tissues respectively. The lower expression levels of AC068338.3 and AL691432.2 were detected with the more advance T-stages.

Conclusions: Our results highlighted some sIRLNRs with significant clinical correlations and demonstrated their monitored and prognostic values for LUAC patients. The results of this study may provide a new perspective for immunological research and immunotherapy strategies.

## INTRODUCTION

With higher mortality rate than breast, colorectal and cervical cancers combined, lung cancer (LCa) becomes a type of common malignant tumor and causes a large proportion of cancer deaths worldwide (accounting for 18.4% of all cancer deaths) [1, 2]. The mainly histological subtypes of LCa are small cell lung cancer (SCLC, accounting for 20%) and non-small cell lung cancer (NSCLC, accounting for 80%) [3]. NSCLC are further classified into adenocarcinoma, squamous cell carcinoma and large cell subtype [3, 4]. With the higher proportion than squamous cell carcinoma and large cell subtype, lung adenocarcinoma (LUAC) which accounting for appropriated 60% of NSCLC is the most common subtype of NSCLC [5–7].

Although radical resection brought hope to patients with LCa, the recurrent rate within 5 years, reaching near 50% [8]. Therefore, increasing number of studies have been exploring more treatment strategies including chemotherapy, radiotherapy, biological targeting and immunotherapy [9]. In recent years, a growing body of immunotherapeutic options, such as PD-1 and PD-L1 blockers, have been approved for LCa, and achieved satisfied outcomes [10, 11]. Additionally, certain studies have revealed the expression levels of immune-related markers such as PD-1 and PD-L1, are remarkably correlated to the effects of immunotherapy [12]. Therefore, the further discoveries of biomarkers of immunotherapeutic effects and prognosis are anticipated.

As the important regulatory factors of gene expression, tumor microenvironment (TME) is closely associated with the tumorigenesis, progression and prognosis [13, 14]. For the past few years, increasing numbers of immune-related genes (IRGs) were identified to help predict prognosis of various cancers [15–17], and some biomarkers in IME have also been documented to assess the immunotherapeutic response [18, 19]. Nevertheless, in the aspect of markers of IRGs, there are still some deficiencies, especially for the prediction of the prognosis of LUAC.

Accumulating evidence has ascertained that long non-coding RNAs (LncRNAs) participate in regulating the immune response and prognosis of tumor [20, 21], and certain immune-related LncRNAs (IRLNRs) are implicated in prognosis evaluation of patients with tumors [22, 23]. Overexpression of lncRNA-MALAT-1 has been explored to associate with the high recurrence rate of hepatocellular carcinoma after liver transplantation [24]. LncRNA-DANCR attenuates SOCS3 transcription and promotes breast cancer progression [25]. TMPO-AS1 was demonstrated to contribute to the tumorigenesis and progression of bladder cancer by regulating mir-98a-5p [26]. Besides, Wei et al. illuminated that TMPO-AS1 promotes the proliferation of LUAC cells via regulating miR-326/SOX12 axis [27]. AL606489.1 was also verified to induce ferroptosis and autophagy in lung cancer [28, 29]. Although increasing studies have clarified the roles of IRLNRs in various tumors, their predicting potential for patients with LUAC remain unclear.

Therefore, we sought to explore the clinical significance of IME and IRLNRs on forecasting the prognosis of LUAC. The study establishes an accurate and appropriate model for prognosis prediction of LUAC patients and contributes to reveal the underlying mechanisms of the forecasting effects of IRLNRs in future researches. More importantly, the results will offer a guiding basis for clinicians.

## MATERIALS AND METHODS

### Clinical samples and cell lines

Tumor and normal adjacent tissues of LUAC patients were obtained from 374 patients admitted to Chongqing University Cancer Hospital, of which 106 T1 stage patients, 192 T2 stage patients, 60 T3 stage patients and 16 T4 stage patients. Human bronchial epithelial cell (HBE) and LUAC cell lines (A549, HCC827, NCI-H1299, NCI-H2228 and NCI-H1975) were obtained from the American Type Culture Collection (ATCC, Virginia, USA). DMEM and 1640 medium, adding 10% fetal bovine serum, 100 μ/ml penicillin and 100 mg/ml streptomycin were used to culture cells. Cells were incubated at 37° C with the 5% concentration of CO_2_.

### Transcriptome data download and pre-processing

We downloaded the transcriptome RNA-sequencing data of LUAC samples from TCGA data portal (https://portal.gdc.cancer.gov/). Besides, we obtained the clinical parameters of these patients and excluded patients with the poor overall survival (less than 30 days) to decrease the non-cancer-specific mortality. These data were current updated in April 07, 2020. The Perl language (http://www.perl.org/) was used to process RNA-seq data and clinical outcomes.

### IRLNRs extraction

IRGs were identified by The Molecular Signatures Database v4.0 (http://www.broadinstitute.org/gsea/msigdb/index.jsp) (Supplementary Files 1, 2). IRGs were utilized to calculate the immune-related score of LUAC through GSEA. The correlations between immune-related score and LncRNAs expression were analyzed through Pearson correlation analysis. IRLNRs need to conform to the standard of |r|>0.4 and *P*<0.001.

### Acquire the survival-related IRLNRs (sIRLNRs)

IRLNRs with correlation of OS were regarded as sIRLNRs in LUAC patients. The sIRLNRs were identified by univariate COX regression analysis (P<0.001). Besides, the sIRLNRs were divided into protective and deleterious portion by Hazard ratio.

### Immune-related risk score model creation

Following multivariate COX regression analysis, the integrated sIRLNRs were regarded as independent prognostic factors to establish the immune-related risk score model (IRRS). IRRS was created based on the expression level of sIRLNRs multiplied by the coefficients of Cox regression. [AC068338.3 * (-0.45635)] + [AL691432.2 * (-0.25455)] + [AL606489.1* (0.210469)] + [TMPO-AS1* (0.473489)] + [AP000695.1* (0.358633)]. We classify patients with LUAC into the high-risk group and the low-risk group using the median score. Besides, we obtained the immune cell infiltration status of LUAC patients, and analyzed their relationships with the IRRS.

### Bioinformatics analysis

Based on the IRRS, the sensitivity and specificity were evaluated via ROC curve. Principal component analysis (PCA) and GSEA was displayed to explore the differential expression levels and phenotypes between patients with various risks. Kaplan-Meier curve was utilized to assess the OS. The independent prognostic factors of LUAC patients were identified by Cox regression analysis. The nomogram was employed to predict the survival probabilities.

### Real-time quantitative PCR

The real-time quantitative PCR (qPCR) was conducted as previous report [30]. Extracted total RNA from tissues and cell lines by Triazole (Invitrogen). The reverse transcribed cDNA was obtained by cDNA Synthesis Kit (Osaka, Japan of TaKaRa). The qPCR was conducted on an ABI 7500 real-time PCR system (Applied Biosystems). The primer sequences are illustrated in Table 1. Each cDNA sample was analyzed three times.

**Table 1:** The primer sequences of AC068338.3 and AL691432.2.

**AC068338.3**	F primer (5’-3’)	AGGCTCGGGAGTGTGATTTG
	R primer (5’-3’)	CTTTGGTGCAGTGTTTCCGG
**AL691432.2**	F primer (5’-3’)	TTTGGACCAAAGGCCTGTGT
	R primer (5’-3’)	CAACTCCACACACATCCCGA
**β-actin**	F primer (5’-3’)	AAACGTGCTGCTGACCGAG
	R primer (5’-3’)	TAGCACAGCCTGGATAGCAAC

Note: F primer, forward primer; R primer, reverse primer.

### Statistical analysis

Statistical analysis was performed by SPSS21.0 software (Chicago, IL, USA) and GraphPad Prism5 (La Jolla, CA, USA). Varieties in clinical parameters were validated using ANOVA, post-hoc test and independent T-test. *P*<0.05 was considered significantly statistical difference.

### Availability of data and materials

Authors can provide all of datasets analyzed during the study on reasonable request.

### Ethics approval and consent to participate

Informed consent forms have been signed by all patients before this study. The research protocol has been approved by the Ethics Committee of Chongqing University Cancer Hospital and is based on the ethical principles of medical research involving human subjects in the Helsinki Declaration.

## RESULTS

### IRLNRs acquisition

Transcriptome RNA-sequencing data and clinical parameters were downloaded from TCGA database. Following that, LncRNAs and mRNAs data were extracted from transcriptome data. We screened 331 IRGs, of which 36 LncRNAs were identified to be the IRLNRs by correlation analysis.

### Correlation between prognosis of LUAC patients and IRLNRs

**Figure 1:**
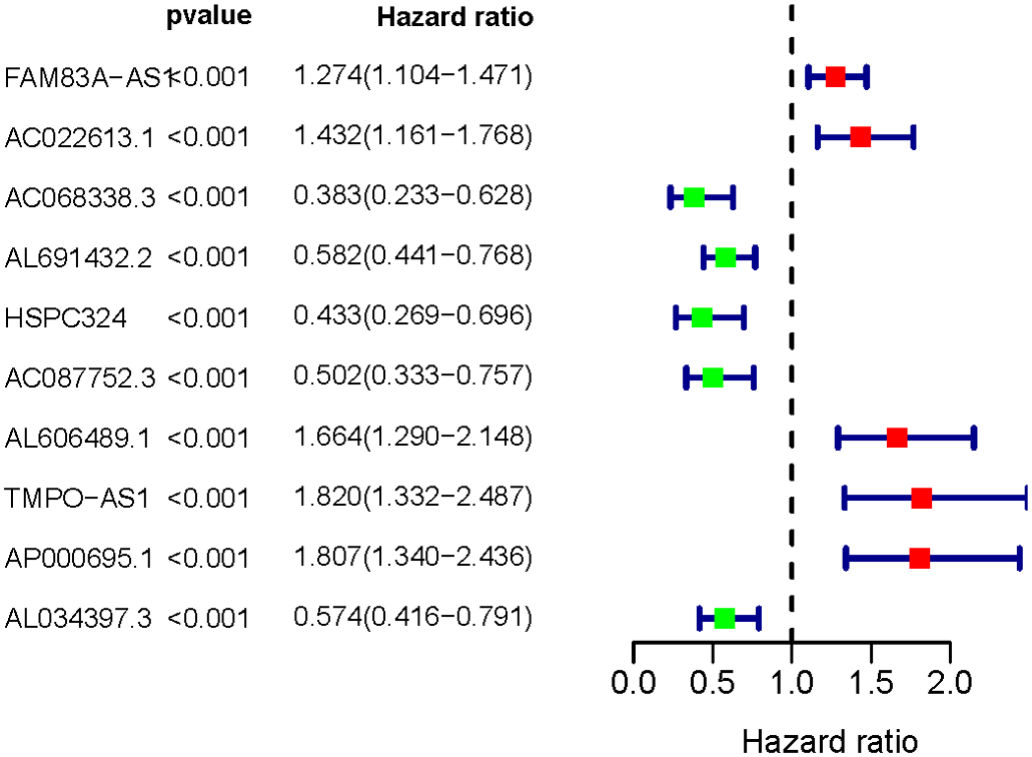
**Survival-related IRLNRs forest plot.** The hazard ratios of survival-related IRLNRs (FAM83A-AS1, AC022613.1, AC068338.3, AL691432.2, HSPC324, AC087752.3, AL606489.1, TMPO-AS1, AP000695.1 and AL034397.3) were illustrated in Forest plot (P<0.001). Red parts represent upregulated sIRLNRs, and green parts represent downregulated sIRLNRs.

Following the univariate COX regression analysis, we then validated 10 IRLNRs which were associated with prognoses of LUAC patients, including FAM83A-AS1, AC022613.1, AC068338.3, AL691432.2, HSPC324, AC087752.3, AL606489.1, TMPO-AS1, AP000695.1 and AL034397.3 (P<0.001). The correlation between the sIRLNRs and prognosis was shown in the forest plot (Figure 1).

### Prognostic features of different risk groups

**Figure 2:**
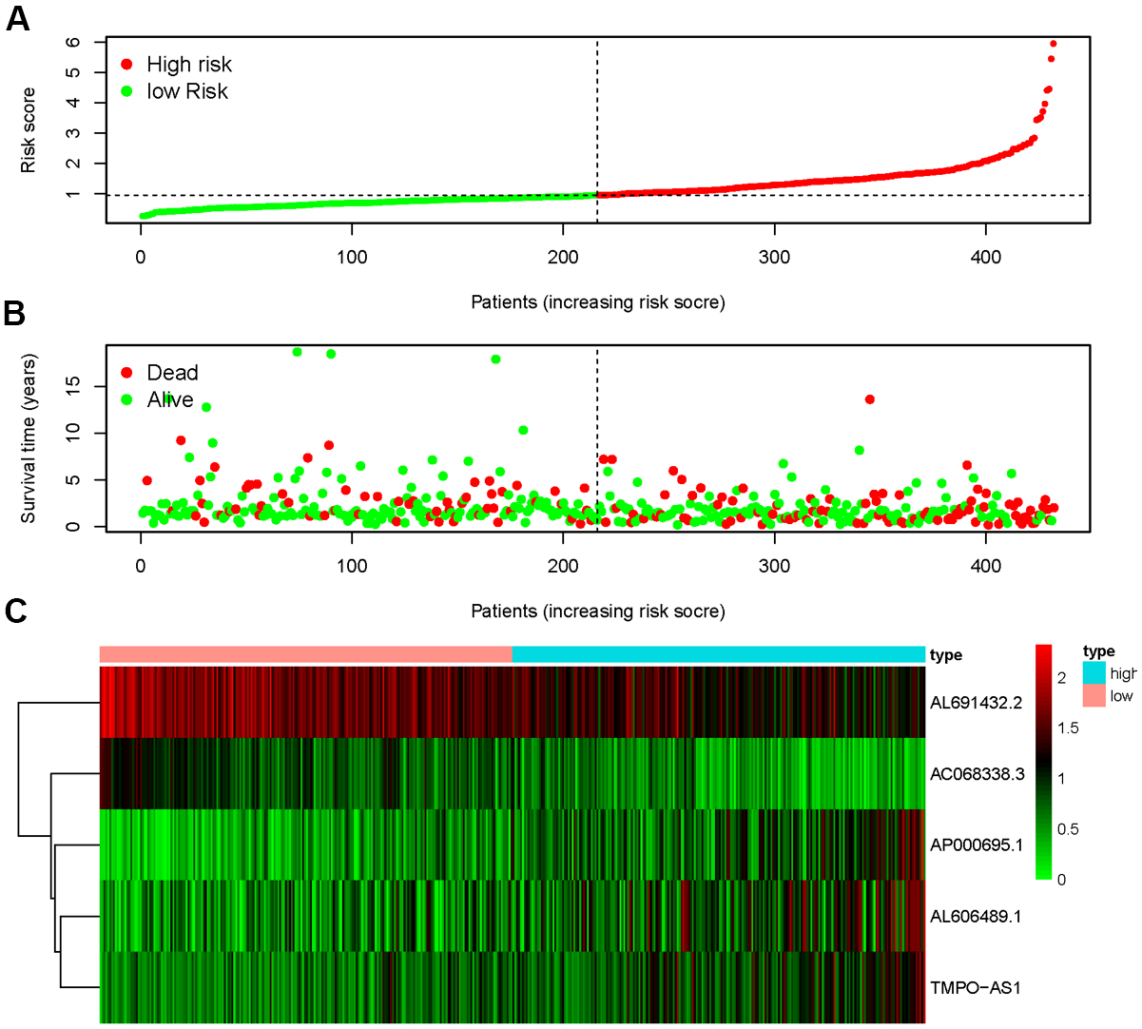
**Immune-related risk score model (IRRS) was established according to sIRLNRs.** The risk score distributions of high-risk group and low-risk group (**A**). Survival status between high-risk group and low-risk group (**B**). The expression levels of sIRLNRs in the heatmap (**C**).

**Figure 3:**
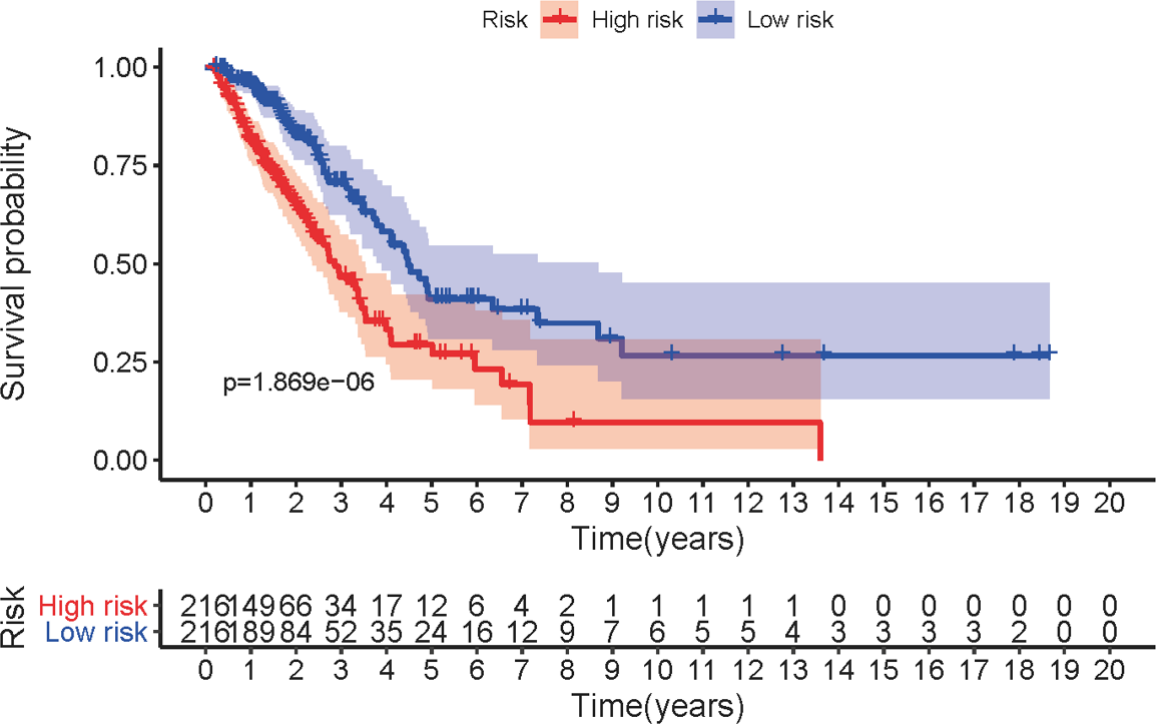
**Survival curve of LUAC patients.** Kaplan-Meier survival curve of OS in high-risk group and low-risk group. The results showed that the high-risk group have the poor prognosis.

The five sIRLNRs (AC068338.3, AL691432.2, AL606489.1, TMPO-AS1 and AP000695.1) among the 10 sIRLNRs were selected by multivariate COX regression analysis, and were utilize to develop the IRRS, by which the LUAC patients were separated into the high-risk group and the low-risk group (Figure 2A). We found that the mortality rate constantly increased with the higher risk score (Figure 2B). With the increase of the risk score, the expression levels of AL606489.1, TMPO-AS1 and AP000695.1 were increased, while AC068338.3 and AL691432.2 were decreased (Figure 2C). Furthermore, as illustrated in Figure 3, compared to the low-risk group, the survival curve of patients in the high-risk group was remarkably lower.

### Correlation between IRRS and clinical features

**Figure 4:**
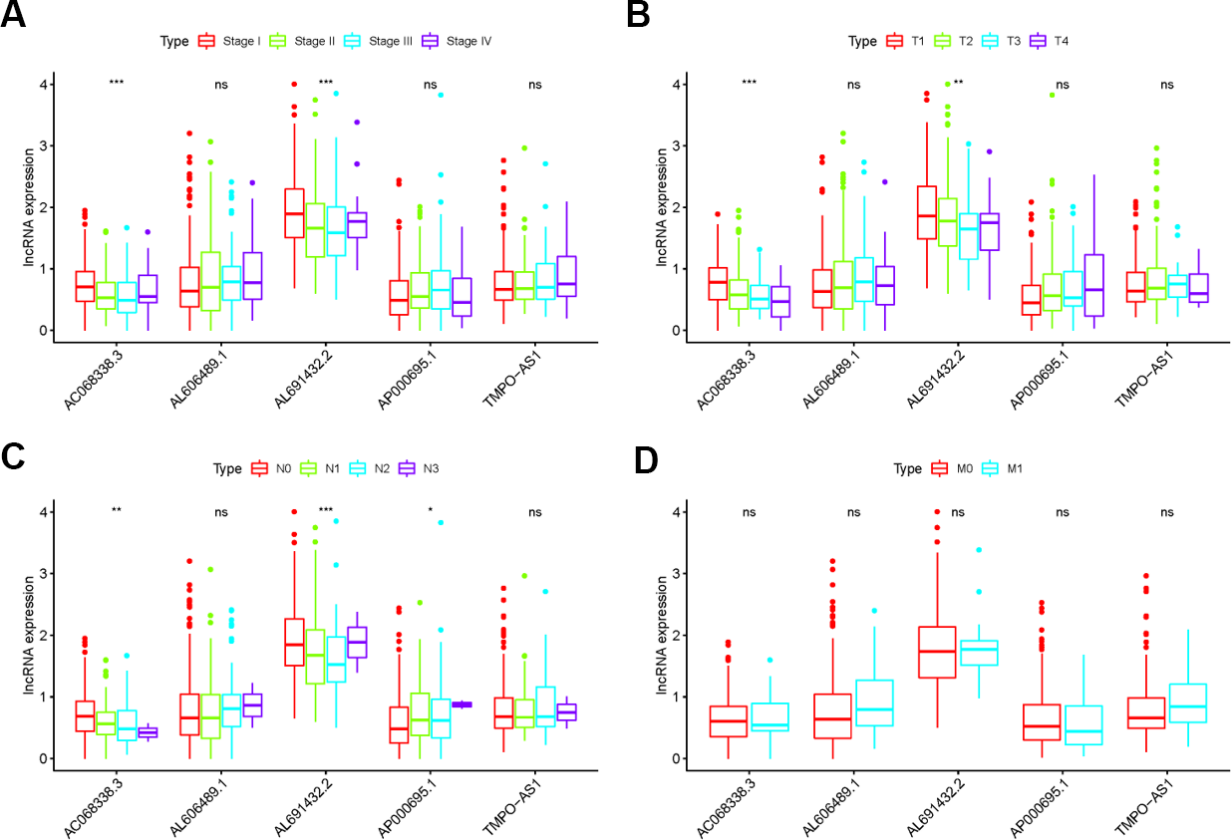
**The relationships between the sIRLNRs and clinical features.** Relationships between sIRLNRs (AC068338.3, AL691432.2, AL606489.1, TMPO-AS1 and AP000695.1) and clinical features. The expression levels of AL606489.1, TMPO-AS1 and AP000695.1 were increased in the more advanced stages (**A**) and T-stages (**B**), while the expression levels of AC068338.3 and AL691432.2 were gradually decreased. The expression levels of AC068338.3 and AL691432.2 were decreased in the more advanced N-stages (**C**), while the expression levels of AP000695.1 were increased. There was no significant difference in various M-stages. (**D**) (***P < 0.001; ** P < 0.01; * P < 0.05; ns: P > 0.05).

**Figure 5:**
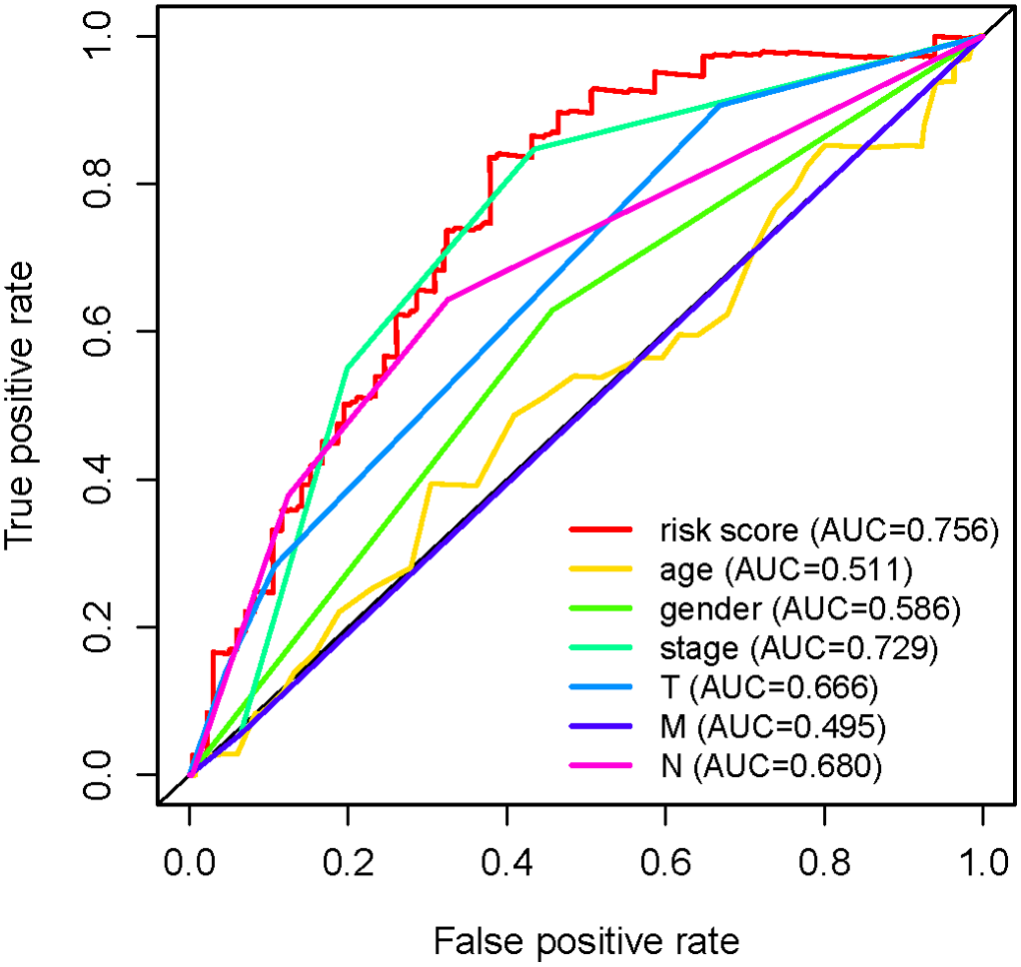
**Receiver operating characteristic (ROC) curve.** The prognostic value of the independent prognostic factors was indicated by ROC curves.

**Figure 6:**
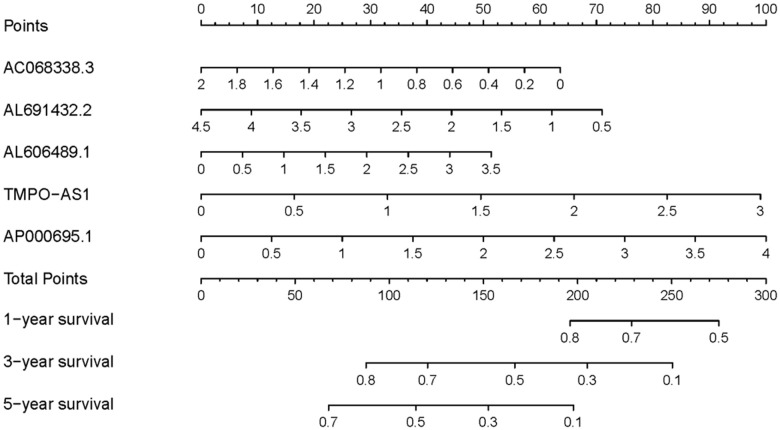
**Nomogram of LUAC patients.** Nomogram was used to predict 1, 3, and 5-year survival rates for LUAC patients.

To explore the clinical significances of the sIRLNRs, we analyzed the relevance of IRRS and clinicopathologic features of LUAC. We found that the expression levels of AC068338.3 and AL691432.2 were lower in LUAC patients with advanced stages and T-stages (Figure 4A, 4B). Besides, the expression of AP000695.1 was higher with the more advanced N-stages, while AC068338.3 and AL691432.2 showed reversed variations (Figure 4C). Additionally, we didn’t observe remarkable differences in various M-stages, and it was probably due to the limited samples size of distant metastasis (Figure 4D). We further displayed univariate COX regression analysis, and we found that stage, T-stage, N-stage, and risk score were prominently associated with OS. Nevertheless, in the multivariate COX regression analysis, only stage and risk score illustrated remarkable correlation with OS (Table 2). Then, the ROC curve was employed to evaluate the accuracy of IRRS. As shown in the Figure 5, the AUC of risk score, age, gender, stage, T-stage, M-stage and N-stage were 0.756, 0.511, 0.586, 0.729, 0.666, 0.495 and 0.680 separately. To further validate the clinical significance and values of IRRS, we drew a nomogram of LUAC by multivariate COX analysis results of the 5 sIRLNRs in the IRRSM (Figure 6). The nomogram will provide another reference for clinical decision-makers to evaluate the prognosis of LUAC patients. These results suggested that risk score is an independent prognostic factor of LUAC.

**Table 2:** Univariate and multivariate analysis of LUAC.

**Variables**	**Univariate analysis**				**Multivariate analysis**			
	**HR**	**HR 95% low**	**HR 95% high**	**P value**	**HR**	**HR 95% low**	**HR 95% high**	**P value**
**Age**	1.001956	0.982501	1.021797	0.845098	1.011486	0.991531	1.031843	0.261273
**Gender**	1.033875	0.711617	1.502069	0.861233	0.923619	0.631409	1.351062	0.682214
**Stage**	1.645457	1.390237	1.947529	6.98E-09	2.03528	1.235723	3.352179	0.005249
**T-stage**	1.589128	1.272484	1.984567	4.40E-05	1.11824	0.875036	1.429038	0.371786
**M-stage**	1.661335	0.888927	3.104904	0.111621	0.32111	0.084868	1.214957	0.094293
**N-stage**	1.784283	1.448821	2.197419	5.06E-08	0.939858	0.611679	1.444112	0.777149
**Risk score**	1.684247	1.423866	1.992243	1.17E-09	1.570501	1.308105	1.885531	1.30E-06

Note: HR, Hazard Ratio.

### The immune status of different risk groups

**Figure 7:**
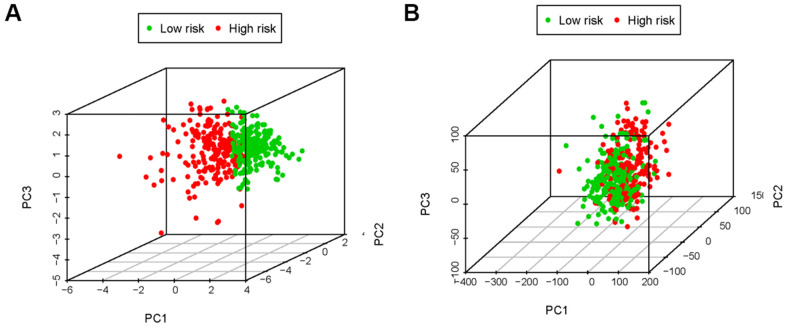
**The principal components analysis (PCA).** The high-risk group and low-risk group tended to express different immune status. PCA among high-risk group and low-risk group based on the immune-related risk gene sets (**A**). PCA among high-risk group and low-risk group based on the genome-wide expression sets (**B**). The results showed that the high-risk group and low-risk group were divided into two parts in immune-related risk gene sets.

In the immune-related risk gene sets, we found that, compared to the low-risk group, the high-risk group obtained the higher immune score (Figure 7A). However, based on the genome-wide expression profiles, the prominent separation between the two risk groups wasn’t observed (Figure 7B).

### The expression of AC068338.3 and AL691432.2 were lower in LUAC cell lines and LUAC patients especially with advanced T-stages

**Figure 8:**
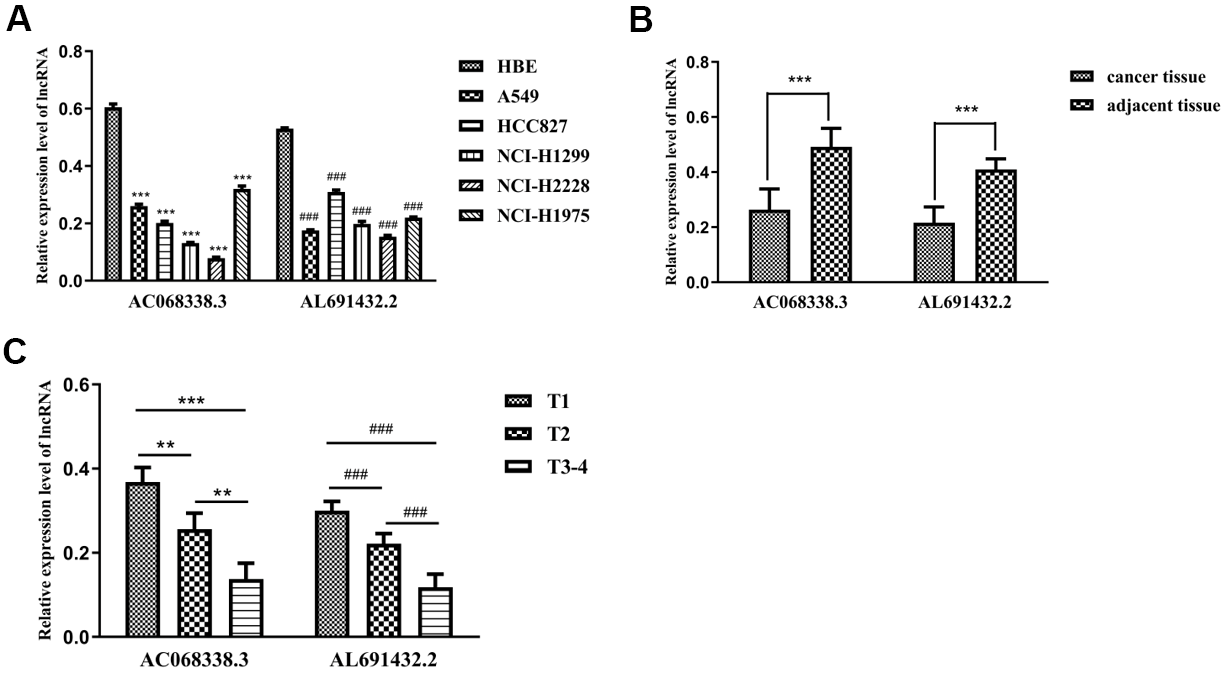
**The expression levels of two sIRLNRs and the relationships with various T-stages.** The results of RT-qPCR of the expression levels of AC068338.3 and AL691432.2 in LUAC cell lines and tumor and adjacent tissues. The expression levels of AC068338.3 and AL691432.2 in HBE were both higher than those in LUAC cell lines, *** represents the significant difference of AC068338.3 compared with HBE (P<0.001), ^###^ represents the significant difference of AL691432.2 compared with HBE (P<0.001) (**A**). Compared with cancer tissues, the expression levels of AC068338.3 and AL691432.2 were both higher in adjacent tissues; *** represents the significant difference of AC068338.3 (P<0.001), ^###^ represents the significant difference of AL691432.2 (P<0.001) (**B**). The expression levels of AC068338.3 and AL691432.2 were both the lowest in LUAC patients of T3-4 stages, and the highest expression levels of AC068338.3 and AL691432.2 were both detected in T1 patients; *** represents the significant difference of AC068338.3 (P<0.001), ** represents the significant difference of AC068338.3 (P<0.01), ^###^ represents the significant difference of AL691432.2 (+<0.001) (**C**).

To validate the expression levels of sIRLNRs *in vivo* and *in vitro*, we examined the expression of AC068338.3 and AL691432.2 in bronchial epithelial cells, different LUAC cell lines, tumor tissues, adjacent normal tissues and tumor samples with different T-stages. We found that, compared to HBE, the expression of AC068338.3 and AL691432.2 were significantly lower in A549, HCC827, NCI-H1299, NCI-H2228 and NCI-H1975 cell lines (Figure 8A). Besides, we detected the remarkably lower expression levels of AC068338.3 and AL691432.2 in tumor tissues than those in adjacent tissues, (Figure 8B) and the expression levels of AC068338.3 and AL691432.2 in T1 and T2 stages were remarkably higher than those in more advanced T-stages (Figure 8C).

## DISCUSSION

Although radical resection is an effective treatment option for LUAC patients, the high recurrence rate after operation and some advanced-stages patients with unresectable tumors are still the great challenges [8]. Therefore, researchers are paying attentions to explore other appropriate treatment strategies for the group of patients. With the in-depth understanding of immune activities in the occurrence, development and prognosis of tumor, a growing body of promising immunotherapies provide new hopes for LUAC patients [31–33]. Up to now, immunotherapy has effectively prolonged the survival time of a large number of patients, but another part of patients showed limited responses [34]. Multiplied studies indicated the individual difference of the gene levels might take responsibility [35, 36].

In the past few years, series of studies have illustrated immune cell infiltration plays the crucial role in the prognosis of patients with LUAC [18, 37–39]. The researchers also highlighted the effect of tumor immune activity on clinical decision-making [40, 41]. Therefore, the roles of gene expression levels on tumor immune activities were deserved to be further explored. Kaiyong Yang reported that Angiotensin II contributed to immunosuppression by induction of PD-L1 expression in NSCLC [42]. Heidi Dvinge indicated that the significant function of miRNA in predicting the prognosis of breast cancer patients [43]. Serum microRNA expression could predict the survival of NSCLC [44]. Overexpression of LncRNA MALAT-1 has been demonstrated to have potential for predicting the recurrence of hepatocellular carcinoma after liver transplantation [24].

Currently, judging the prognosis of patients with tumor by IRGs has also become a research focus [45, 46]. Song Qian has demonstrated IRGs could be served as biomarkers to predict the prognosis of LUAC [47]. In the field of immune microenvironment, emerging evidence has suggested that lncRNAs are closely involved in the regulation of immune autoimmunity and function [48]. LncRNAs have been ascertained to participate in regulating various important immune processes, such as the production of inflammatory mediators, cells differentiation and migration [49]. Xu et al. reported that SATB2-AS1 can regulate the expression of TH1 chemokine and the density of immune cells in CRC to inhibit the progression of CRC [50]. But the mechanism of IRLNRs on the prognosis of patients with LUAC has not been fully elucidated.

In this study, we analyzed TCGA LncRNAs data of 533 patients with LUAC, and screened 311 IRGs, of which 36 IRLNRs were identified as IRLNRs. Besides, we found 10 IRLNRs with prominent relevance to OS. Five IRLNRs were further identified to create an IRRS, by which LUAC patients with different risk scores were distinguished. Following the multivariate COX analysis, we found that risk score is an independent prognostic factor to assess the prognosis of LUAC patients. Given that the low purity of tumor and the infiltration of immune cell components were closely associated with the tumor development, immunophenotype and prognosis [51, 52], we therefore used PCA to explore the differential distribution of different risk groups, and we observed the lower immune score in patients with the lower risk. Besides, neutrophil cells were enriched in the high-risk group, but B cells were enriched in the low-risk group. Therefore, we think that IRRS is significantly related to the immune status of LUAC patients.

Additionally, to increase the reliability of clinical evidences, a great number of patients with LUAC were recruited in the present study. To further validate the correlation between sIRLNRs and tumor clinical features, we examined the expression of AC068338.3 and AL691432.2 *in vitro* and *in vivo.* These results suggested that IRRS based on the five sIRLNRs can help doctors to identify LUAC patients with the similar clinicopathologic and molecular characteristics, and then contribute to the more precise treatment strategies.

We have verified the expression levels of some sIRLNRs (AC068338.3 and AL691432.2) of IRRS in LUAC cell lines and tumor tissues and adjacent tissues of LUAC patients. As for other 3 lncRNAs which were not further validated in the study, we found that TMPO-AS1 plays remarkable roles in promoting tumorigenesis and development of bladder cancer, liver cancer, cervical cancer and lung cancer [15, 26, 53]. In Mu’s study, TMPO-AS1 was demonstrated to promote lung adenocarcinoma progression by targeting miR-383-5p [54]. Besides, AL606489.1 was demonstrated to be regulate ferroptosis and autophagy [28, 29]. Few reports have studied the role of AP000695.1 in cancer, so more focus need to be turned toward the effect and underlying mechanism of AP000695.1 in LUAD.

## CONCLUSIONS

In this study, we identify and validate the LUAC-related sIRLNRs with prominent clinical significances, and create a reliable risk assessing model based on the sIRLNRs to assess prognosis of LUAC patients. The results will offer a novel perspective for immune-related studies and immunotherapeutic strategies of LUAC.

## Supplementary Material

Supplementary File 1

Supplementary File 2
