# Supplementary material for: A novel immune-related long non-coding RNAs risk model for prognosis assessment of lung adenocarcinoma
Source: Aging (Albany NY). 2021 Dec 14;13(23):25550–63. doi: 10.18632/aging.203772 (PMC8714149; doi:10.18632/aging.203772)
Supplement: Supplementary File 1 [file aging-13-203772-s001.docx]

**Supplementary File 1: The screened data in the immune system process M13664 of molecular signatures database.**

IMMUNE_SYSTEM_PROCESS

> Genes annotated by the GO term GO:0002376. Any process involved in the development or functioning of the immune system, an organismal system for calibrated responses to potential internal or invasive threats.

ACIN1

ACKR2

ACKR4

ACVR1B

ACVR2A

ADGRE5

AIM2

AIMP1

AKT1

ALAS2

ANXA11

APLN

APOA1

APOA2

APOA4

APOBEC3F

APOBEC3G

AQP9

ARHGDIB

ATP6V0A2

AZU1

BCAR1

BCL10

BCL2

BLNK

BNIP3

BNIP3L

BST1

BST2

C1QBP

C2

C5AR1

CADM1

CALCA

CARTPT

CCL18

CCL19

CCL2

CCL20

CCL21

CCL22

CCL23

CCL24

CCL25

CCL26

CCL27

CCL4

CCL5

CCR1

CCR2

CCR4

CCR5

CCR6

CCR8

CCR9

CD164

CD1D

CD2

CD22

CD24

CD274

CD276

CD28

CD34

CD3D

CD3E

CD4

CD40LG

CD47

CD7

CD74

CD79A

CD79B

CD83

CD86

CD96

CDC42

CDK6

CEACAM8

CEBPB

CEBPG

CFHR1

CHST4

CHUK

CIITA

CKLF

CLEC7A

CMKLR1

CNIH1

CNR2

COLEC12

CRHR1

CRTAM

CSF1

CST7

CTLA4

CTSC

CTSE

CTSG

CTSS

CTSW

CX3CL1

CXCL12

CXCL13

CXCL8

CXCR2

CXCR4

DCSTAMP

DEFA1

DEFB1

DEFB103A

DEFB118

DEFB127

DEFB4A

DMBT1

DOCK2

DPP4

DPP8

DYRK3

EBI3

ELF4

ELP1

ERAP2

EREG

ETS1

FCAR

FCGR1A

FCGR2B

FCGR3A

FCGR3B

FCGRT

FCN1

FCN2

FOXO3

FOXP3

FTH1

FYB1

FYN

GBP2

GEM

GLMN

GPI

GPR183

GPR65

GTPBP1

GZMA

HAMP

HCLS1

HDAC4

HDAC5

HDAC7

HDAC9

HELLS

HLA-DRB3

HRH2

ICOSLG

IFI16

IFI6

IFITM2

IFITM3

IFNK

IFNL1

IFNLR1

IGSF6

IK

IKBKG

IL10

IL10RB

IL12A

IL12B

IL15

IL16

IL17A

IL17B

IL18

IL18BP

IL1R2

IL2

IL21

IL27

IL27RA

IL2RA

IL2RG

IL31RA

IL32

IL4

IL4R

IL6

IL6R

IL6ST

IL7

IL7R

INHA

INHBA

INS

IRAG2

IRF8

ITGB2

JAG2

KAT6A

KAT8

KIR2DL1

KIR2DL3

KIRREL3

KMT2A

KRT1

LAT

LAT2

LAX1

LCK

LCP2

LDB1

LIG1

LIG3

LILRB2

LST1

LTB4R

LTF

LY75

LY86

LYN

MADCAM1

MAFB

MAL

MALT1

MAP3K7

MAP4K1

MAP4K2

MBL2

MBP

MIA3

MLF1

MMP9

MNX1

MR1

MS4A1

MS4A2

MYH9

NCF4

NCK1

NCK2

NCOA6

NCR1

NFAM1

NFIL3

NHEJ1

NLRC3

NOTCH2

NOTCH4

OPRD1

OPRK1

PAX5

PDCD1

PF4

POU2AF1

POU2F2

PRELID1

PREX1

PRG3

PRKRA

PRL

PSMB10

PTAFR

PTGDR2

PTGER4

PTPRC

PYDC1

RAB3D

RAG1

RASGRP4

RFX1

RGS1

RPS19

RSAD2

RUNX1

S1PR4

SAA1

SART1

SCG2

SCIN

SECTM1

SEMA3C

SEMA4D

SEMA7A

SFTPD

SIRPG

SIT1

SKAP1

SLA2

SNRK

SOCS5

SOD1

SP2

SPACA3

SPI1

SPINK5

ST6GAL1

SYK

TAPBP

TARBP2

TAZ

TBX1

TCF12

TCF7

TENM1

TGFB1

TGFB2

THY1

TLR4

TLR7

TLR8

TNFAIP1

TNFRSF14

TNFRSF4

TNFSF13

TPD52

TRAF2

TRAF6

TRAT1

TREM1

TREM2

TRIM22

UBE2N

VIPR1

VTN

WAS

XBP1

YTHDF2

ZAP70

ZBTB16

ZEB1

ZNF675
