# Supplementary material for: A novel immune-related long non-coding RNAs risk model for prognosis assessment of lung adenocarcinoma
Source: Aging (Albany NY). 2021 Dec 14;13(23):25550–63. doi: 10.18632/aging.203772 (PMC8714149; doi:10.18632/aging.203772)
Supplement: Supplementary File 2 [file aging-13-203772-s002.docx]

**Supplementary File 2. The screened data in the immune response M19817 of molecular signatures database.**

IMMUNE_RESPONSE

> Genes annotated by the GO term GO:0006955. Any immune system process that functions in the calibrated response of an organism to a potential internal or invasive threat.

ACKR2

ACKR4

ADGRE5

AIM2

ANXA11

APLN

APOA1

APOA2

APOA4

APOBEC3F

APOBEC3G

AQP9

ARHGDIB

ATP6V0A2

BCAR1

BCL10

BCL2

BLNK

BNIP3

BNIP3L

BST1

BST2

C1QBP

C2

C5AR1

CADM1

CCL18

CCL19

CCL2

CCL20

CCL21

CCL22

CCL23

CCL24

CCL25

CCL26

CCL27

CCL4

CCL5

CCR1

CCR2

CCR4

CCR5

CCR6

CCR8

CCR9

CD164

CD1D

CD22

CD274

CD28

CD40LG

CD7

CD74

CD79A

CD79B

CD83

CD86

CD96

CEACAM8

CEBPB

CEBPG

CFHR1

CHST4

CHUK

CIITA

CMKLR1

CNIH1

CNR2

COLEC12

CRHR1

CRTAM

CST7

CTLA4

CTSC

CTSG

CTSS

CTSW

CX3CL1

CXCL12

CXCL13

CXCR4

DEFA1

DEFB1

DEFB103A

DEFB118

DEFB127

DEFB4A

DMBT1

DPP4

DPP8

EBI3

ELP1

EREG

ETS1

FCAR

FCGR1A

FCGR2B

FCGR3A

FCGR3B

FCGRT

FCN1

FCN2

FOXP3

FTH1

FYB1

FYN

GBP2

GEM

GPI

GPR183

GPR65

GTPBP1

GZMA

HAMP

HLA-DRB3

HRH2

IFI6

IFITM2

IFITM3

IFNK

IFNL1

IFNLR1

IGSF6

IK

IKBKG

IL10

IL10RB

IL12A

IL12B

IL15

IL16

IL17A

IL17B

IL18

IL18BP

IL1R2

IL2

IL27

IL27RA

IL2RA

IL2RG

IL32

IL4

IL4R

IL6

IL6R

IL6ST

IL7

IL7R

IRF8

KIR2DL1

KIR2DL3

KRT1

LAT

LAT2

LAX1

LCP2

LILRB2

LTB4R

LTF

LY75

LY86

MADCAM1

MALT1

MAP3K7

MAP4K2

MBL2

MBP

MNX1

MR1

MS4A1

MS4A2

NCF4

NCR1

NFAM1

NFIL3

OPRD1

OPRK1

PAX5

PDCD1

POU2AF1

POU2F2

PRELID1

PRKRA

PSMB10

PTAFR

PTGDR2

PTGER4

PTPRC

PYDC1

RAG1

RFX1

RGS1

RSAD2

S1PR4

SECTM1

SEMA3C

SEMA4D

SEMA7A

SFTPD

SKAP1

SLA2

SOCS5

SP2

SPINK5

ST6GAL1

TAPBP

TARBP2

TCF12

TCF7

TENM1

TGFB1

TGFB2

THY1

TLR7

TLR8

TNFAIP1

TNFRSF14

TNFRSF4

TNFSF13

TRAF2

TRAF6

TRAT1

TREM1

TREM2

TRIM22

UBE2N

VIPR1

VTN

WAS

XBP1

YTHDF2

ZAP70

ZEB1
